# Smartphone-based multi-contrast microscope using color-multiplexed illumination

**DOI:** 10.1038/s41598-017-07703-w

**Published:** 2017-08-08

**Authors:** Daeseong Jung, Jun-Ho Choi, Soocheol Kim, Suho Ryu, Wonchan Lee, Jong-Seok Lee, Chulmin Joo

**Affiliations:** 1 Yonsei University, School of Mechanical Engineering, Seoul, 03722 Republic of Korea; 2 Yonsei University, School of Integrated Technology & Yonsei Institute of Convergence Technology, Incheon, 21983 Republic of Korea

## Abstract

We present a portable multi-contrast microscope capable of producing bright-field, dark-field, and differential phase contrast images of thin biological specimens on a smartphone platform. The microscopy method is based on an imaging scheme termed “color-coded light-emitting-diode (LED) microscopy (cLEDscope),” in which a specimen is illuminated with a color-coded LED array and light transmitted through the specimen is recorded by a color image sensor. Decomposition of the image into red, green, and blue colors and subsequent computation enable multi-contrast imaging in a single shot. In order to transform a smartphone into a multi-contrast imaging device, we developed an add-on module composed of a patterned color micro-LED array, specimen stage, and miniature objective. Simple installation of this module onto a smartphone enables multi-contrast imaging of transparent specimens. In addition, an Android-based app was implemented to acquire an image, perform the associated computation, and display the multi-contrast images in real time. Herein, the details of our smartphone module and experimental demonstrations with various biological specimens are presented.

## Introduction

Microscopes constitute one of the most commonly employed equipment in biology and medicine^1^. Continued advances in microscopy have introduced many new modalities; however, the relatively large sizes of microscopes, along with their complexity and cost, often limit the utility of these devices in general population and diverse field settings. Recent advances in smartphone technology have made a significant impact on microscopy^2–9^, transforming the bulky and expensive microscope into a portable and low-cost device. Smartphones are equipped with high computing power, high-performance sensors, and wireless network connectivity. Further, the camera modules in smartphones, in particular, employ state-of-the-art image sensors with small pixel sizes and high pixel counts. Microscopes using these built-in camera modules facilitate compact and portable imaging, providing a wide range of opportunities with regard to education, telemedicine, and remote diagnostics.

Diverse imaging modalities have thus been implemented on the smartphone platform. Among the many modalities, label-free imaging on a portable platform is particularly useful in resource-limited settings, as it does not require expensive and time-consuming sample preparation procedures. Bright-field (BF) microscopy is the simplest and most common form of label-free microscopy. However, this technique is unsuitable for the observation of translucent specimens such as unlabeled cell monolayers and thin tissue sections, as these specimens do not exhibit strong attenuation in visible light. Dark-field (DF)^10, 11^ and phase contrast microscopes^12, 13^, on the other hand, offer higher contrast and detailed structural information of unlabeled specimens compared to BF imaging. Therefore, these modalities are extensively utilized for various studies in laboratory settings.

Recently, Philips *et al*.^14^ demonstrated a multi-contrast microscope on a smartphone platform capable of generating BF, DF, and differential phase contrast^15–20^ (DPC) images. In this method, a programmable light-emitting-diode (LED) array in a domed arrangement illuminates the specimen at different angles. Synchronized image acquisition with the smartphone camera module and subsequent computation then facilitate BF, DF, and DPC imaging. The application of this method, however, requires synchronized operation of the external LED light source and image sensor, corresponding to relatively high implementation complexity. For multi-contrast imaging, this method also requires sequential acquisition of multiple images with different illumination patterns, necessitating a minimum of three images in order to obtain BF, DF, and DPC images.

Here, we present a simpler and more cost-effective strategy for smartphone-based multi-contrast imaging. The proposed method is capable of producing BF, DF, and DPC images *in a single shot* and thus, the synchronized operation of the LED array and smartphone camera module is not required. The enabling device consists of a lightweight (~250 g) opto-mechanical smartphone attachment and a custom-developed Android application (“App”) for acquisition, computation, and display of the imaging results. The basic strategy of our method involves color-coded LED illumination, in which red, green, and blue colors correspond to different illumination angles. Image acquisition with a built-in smartphone color image sensor, and subsequent decomposition and computation using the images in each color channel produces BF, DF, and DPC images in a single shot.

Here, we describe the implementation of our smartphone-based multi-contrast microscope and demonstrate its imaging capability by presenting multi-contrast images of various biological specimens. Having a compact design and exhibiting robust performance, our proposed microscope is likely to constitute an ideal imaging tool for educational purposes as well as diagnostics in resource-limited settings.

## Results and Discussion

### Color-coded LED microscopy (cLEDscope)

**Figure 1:**
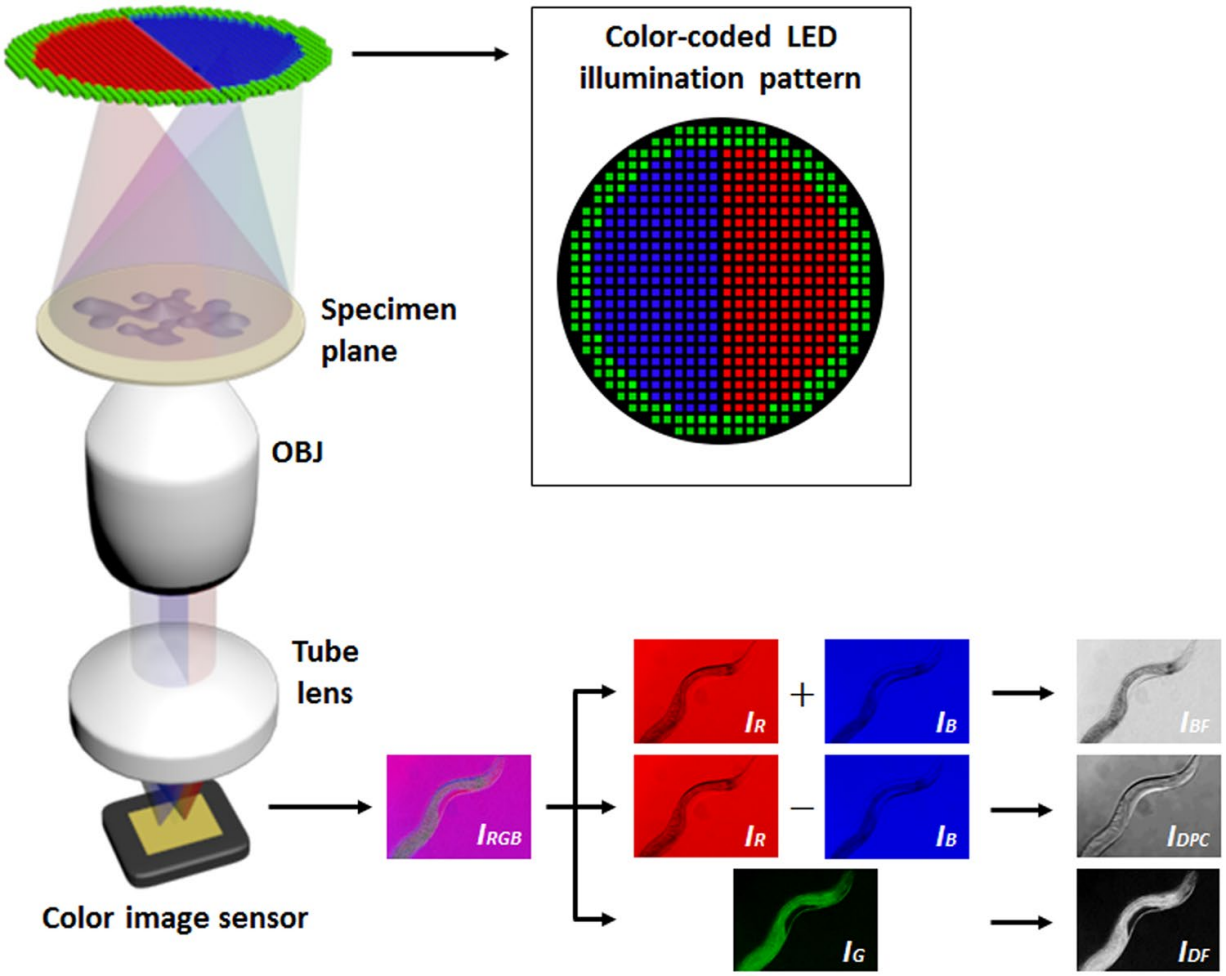
Operating principle of color-coded LED microscopy. Patterned illumination with a color LED array and subsequent computation enable acquisition of bright-field (BF), dark-field (DF), and differential phase-contrast (DPC) images in a single shot. Summation of the images in red (R) and blue (B) colors produces BF images. The image in green (G) corresponds to the DF image. The DPC image is obtained by evaluating the difference between the images in red and B, with subsequent division of the difference by the BF image. OBJ: Objective lens.

Operation of our mobile multi-contrast microscope is based on the previously demonstrated microscopy scheme termed “color-coded LED microscopy (cLEDscope)^21^”. (Fig. 1) In cLEDscope, a color LED array is used as the light source, and is placed at a certain distance from the specimen plane, such that its position is approximately located in the Fourier plane of the specimen plane. Light transmitted through the specimen is then imaged by a color image sensor.

In order to obtain BF, DF, and DPC images in a single shot, the LED array is patterned as illustrated in Fig. 1. The pattern consists of two half circles allocated to red and blue, while the outer circle is assigned to green. The circle size is determined by the numerical aperture (NA) of the detection objective (OBJ). Therefore, the angles of the green LEDs correspond to illumination angles larger than the maximum acceptance angle of the microscope.

For image reconstruction, the color image $({I}_{RGB})$ is decomposed into three images of red $({I}_{R})$, green $({I}_{G})$, and blue $({I}_{B})$, and computations performed using Eqs 1–3 yield BF $({I}_{BF})$, DF $({I}_{DF})$, and DPC $({I}_{DPC})$ images. Summation of ${I}_{R}$ and ${I}_{B}$ is equivalent to recording the image with full circular illumination, thereby producing a BF image (Eq. 1). On the other hand, the difference between ${I}_{R}$ and ${I}_{B}$ arises from the specimen phase gradient, which alters the transmitted light path and shifts the LED light pattern in the pupil plane proportional to the phase gradient. Thus, the light transmitted through the aperture differs for the red and blue colors, and this difference yields the image contrast in the DPC images. Note that the normalization operation in Eq. 2 minimizes intensity-related noises. The DPC image formation can be analytically described by phase-gradient transfer functions, as detailed in several studies^22, 23^. The DPC images in other directions can be obtained by changing the LED array pattern accordingly. It should be noted that the DPC images in multiple directions can be utilized to compute quantitative phase image of the specimen. Lee *et al*.^21^ indeed obtained two DPC images by recording the images with the LED pattern in Fig. 1 at 0 and 90 degrees. The obtained DPC images were then used to evaluate quantitative phase information. Philips *et al*.^20^ and Lee *et al*.^24^ also demonstrated single-exposure quantitative phase imaging via color-multiplexed illumination, albeit with different color-encoding strategies and computation algorithms. In our mobile cLEDscope, the LED illumination in Fig. 1 was employed to achieve single-shot BF, DF and DPC imaging; the red and blue LEDs were patterned inside the circle that corresponds to the detection NA, while green LEDs were outside the circle. The image in the green channel then results from the scattered light obtained under oblique illumination with angles larger than the objective NA (Eq. 3). Computations based on the three-color images therefore yield BF, DF, and DPC images in a single shot.

1 $${I}_{BF}={I}_{R}+{I}_{B},$$

2 $${I}_{DPC}=({I}_{R}-{I}_{B})/{I}_{BF},$$

3 $${I}_{DF}={I}_{G}$$

### cLEDscope smartphone module

**Figure 2:**
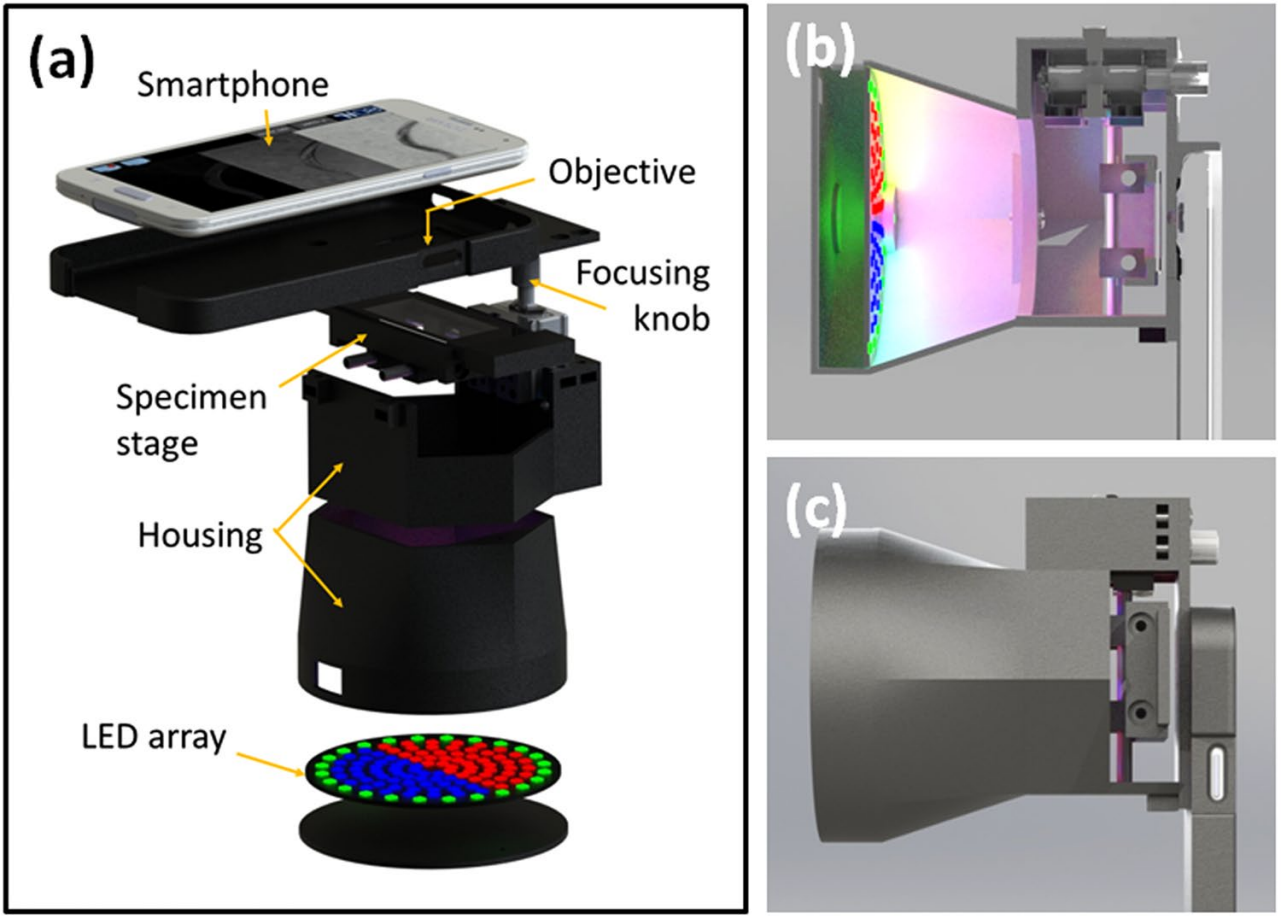
cLEDscope module for multi-contrast imaging. (**a**) Smartphone module system arrangement. (**b,c**) Smartphone cLEDscope, housing-free and covered, respectively.

In order to enable cLEDscope operation in a smartphone without any modification of its hardware, we designed a portable and lightweight cLEDscope module, as depicted in Fig. 2. The cLEDscope module was designed for application on a Samsung Galaxy S5 smartphone; however, it can be readily adopted for operation with other smartphones. The module features a patterned color LED array, manual specimen stage, focusing knob, and miniature objective. The circular LED panel is set to 80 mm in diameter, which matches to the detection numerical aperture (NA) in our system (~0.25). The LED array is patterned as depicted in Fig. 1, so that inner circle is divided in half and allocated to the red and blue LEDs, whereas the green LEDs are mounted along the perimeter of the circular pattern. The LED array is powered by a separate battery, and can be operated with a switch in the module. Once the module is installed on the smartphone, users can load and position the specimen via the stage along three-dimensional axes. The focusing knob also provides fine control of the specimen axial position. The specimen stage is located at 75–80 mm away from the LED array. The light transmitted through the specimen is collected by a miniature objective, and imaged onto the smartphone image sensor via the built-in lens. The optical arrangement is detailed in the next Section.

**Figure 3:**
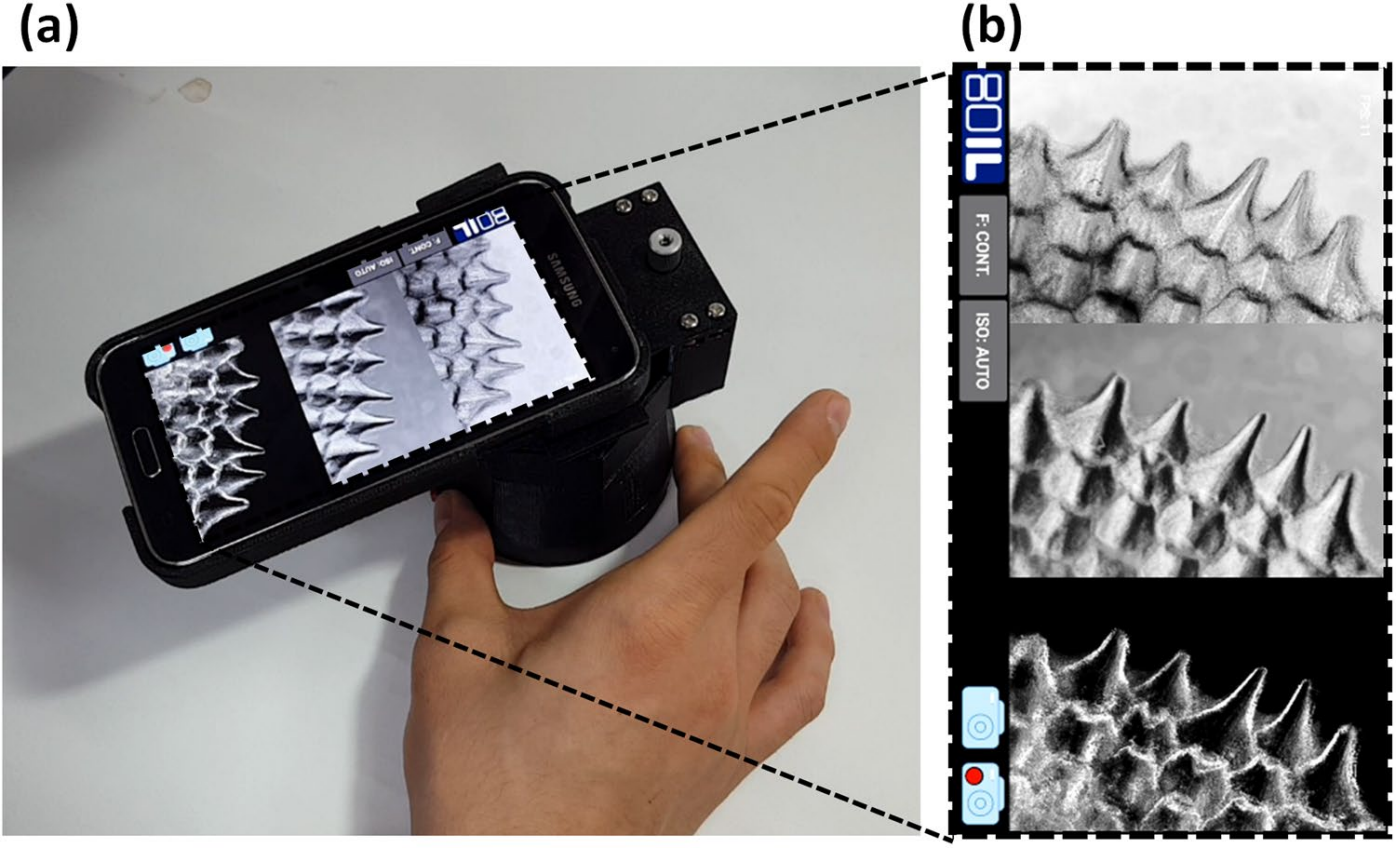
Operation of smartphone-based cLEDscope.

Figure 3(a) shows a photograph of the assembled cLEDscope module, which has dimensions of 84 × 110 × 175 mm^3^ and weighs ~250 g. For cLEDscope imaging, the module is attached to a smartphone and the LED power is activated. Then, the cLEDscope App is operated using the smartphone OS. The cLEDscope App was developed to acquire, compute, and display the computed images of a specimen (Methods and materials for details). Figure 3(b) shows screenshots of the App output. App displays BF, DPC, and DF images in real time, and also captures high-resolution images or records videos. In addition, the camera focus mode and exposure time (i.e., the ISO) can be controlled. App in operation can be seen in Supplementary Video 1.

### Imaging setup

For efficient BF and DPC imaging, a specimen should be evenly illuminated by both red and blue LED light, and the transmitted light of each color should also reach and be detected by the image sensor. This establishes a requirement for the objective lens, given a built-in smartphone camera lens. The built-in camera lens is typically constructed from multiple refractive elements and characterized by a wide angle of view, albeit a small aperture.

**Figure 4:**
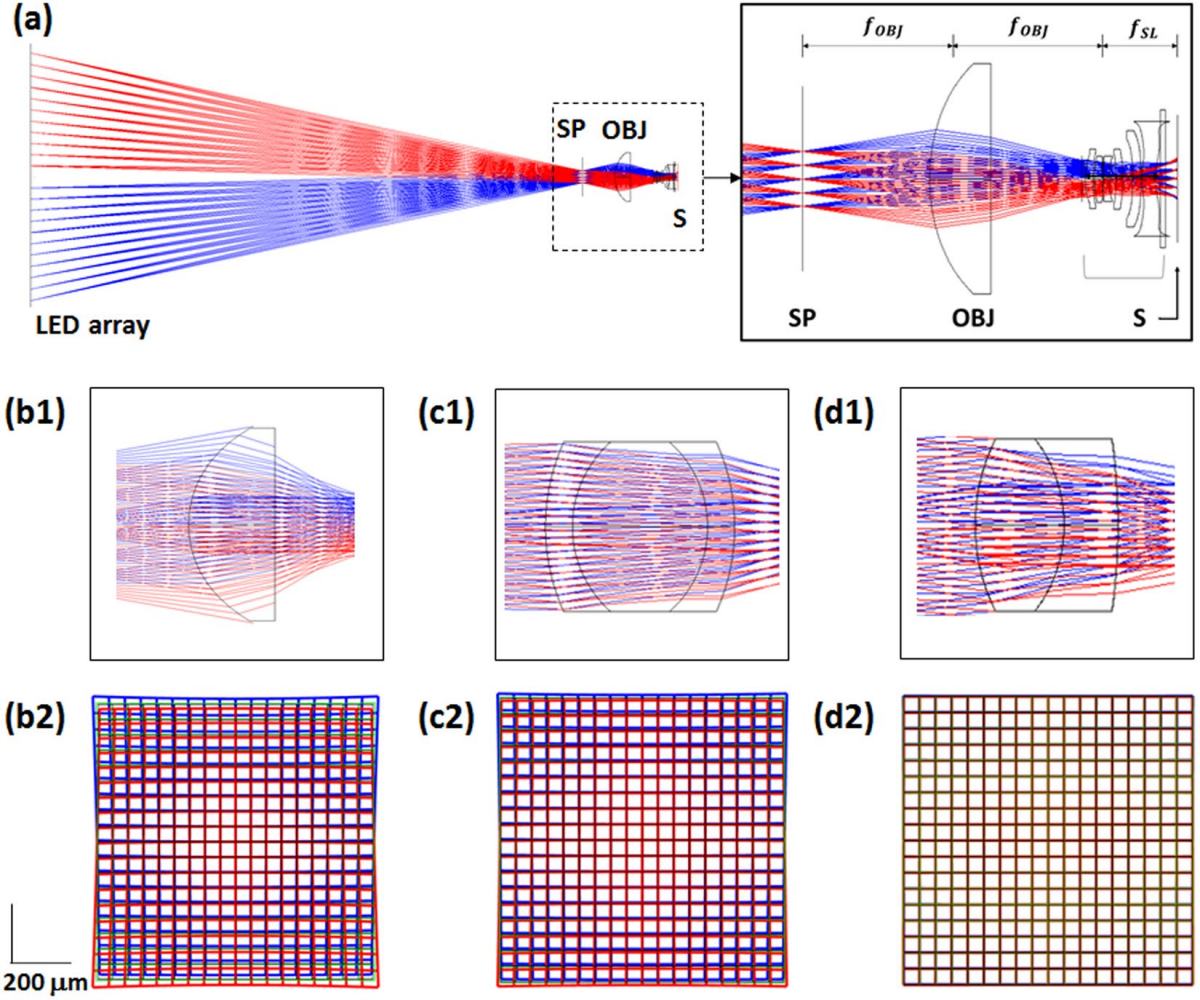
(**a**) ZEMAX model of optical arrangement in mobile cLEDscope. The LED array is positioned a certain distance from the specimen plane (SP). The transmitted light is collected by the objective (OBJ) and detected by a smartphone image sensor (S). The built-in lens in front of the image sensor serves as a tube lens. Inset: Magnified view of imaging setup. (**b1−d1**) Ray trajectories of red and blue light through singlet aspheric, triplet achromatic, and doublet achromatic aspheric lenses, respectively. (**b2−d2**) Corresponding image field distortion maps.

We constructed a ZEMAX model of our optical configuration (Fig. 4(a)), and evaluated several commercial miniature lenses as the objective. For analysis, the specifications for a Samsung camera lens with an *f*-number of 2.2 (focal length = 4.74 mm; lens diameter = 6.2) were obtained from the published patent^25^. The LED array was positioned 76 mm from the specimen plane, and the miniature OBJ and the built-in lens were arranged to form a 3-*f* imaging system, as illustrated in Fig. 4a (see the inset for a magnified view of the 3-*f* setup). The advantage of the 3-*f* configuration over a 4-f alternative is that this arrangement offers the largest acceptance of red and blue light from the light source through the built-in smartphone lens, by projecting the image of the light source onto the smartphone lens plane. The optical magnification is then determined by the focal length ratio of the built-in camera lens to the objective. The light ray trajectories and image field distortion maps for three representative lenses are shown in Fig. 4(b–d). The lenses are singlet aspheric, triplet achromatic, and doublet achromatic aspheric lenses, and detailed information of the lenses is provided in Table S1. Note that the singlet aspheric lens exhibited the largest aberration, producing significantly distorted images for each color (Fig. 4(b2)). The triplet (Fig. 4(c)) and doublet (Fig. 4(d)) cases were relatively well corrected; however, the doublet was found to be superior to the other lenses, as it provided the smallest field distortion. Hence, the doublet achromatic aspheric lens was chosen for use in our prototype.

The optical magnification achieved with the doublet objective was estimated to be 1.3. As the Galaxy S5 image sensor has dimensions of 8.51 × 4.79 mm^2^ and the screen is 5.1-in diagonal with a 16:9 aspect ratio, the unzoomed image has an effective magnification of 17.2x. One should also note that the sensor has a higher pixel count than the screen (i.e., 5312 × 1988 vs. 1920 × 1080, respectively), suggesting that the image can be digitally enlarged on the screen if full resolution magnification information is required. For a sensor pixel pitch of 1.13 μm and a display pixel pitch of 432 ppi, a 1:1 sensor-to-screen pixel ratio is equivalent to an effective magnification of 53x.

### Image distortion correction

Despite careful selection of the objective, optical aberrations and discrepancy between the smartphone camera lens in the ZEMAX analysis and the actual camera lens can induce color-dependent image distortion. In order to compensate for the image distortion, we first examined the image distortion for each color illumination and corrected the image distortion based on a camera calibration strategy previously suggested by Zhang^26^. Details of the distortion correction are provided in Methods and Materials.

**Figure 5:**
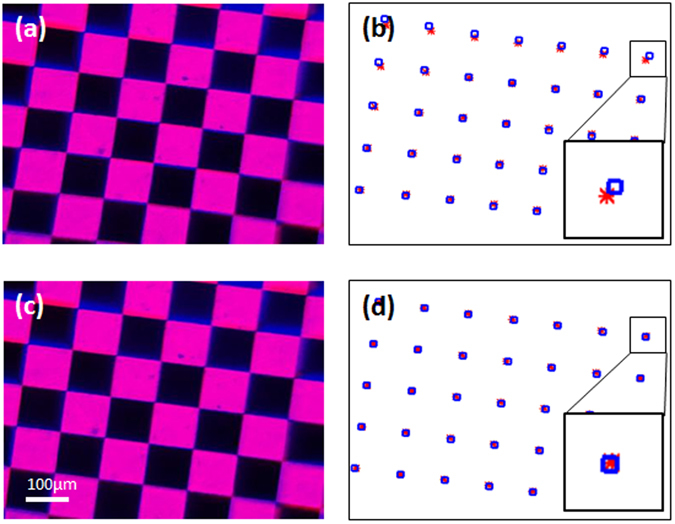
Image distortion correction. (**a,b**) Color image of a binary grid pattern and node positions found in the distortion-uncorrected case. The red stars and blue squares represent the node positions for red and blue LED illuminations, respectively. It is apparent that the node positions are not co-located for each color, especially in the edge of the field of view. (**c,d**) Image of the grid pattern and node positions after the distortion correction. The node positions for each color are well co-located. Insets in (**c**) and (**d**) are the magnified view of the node positions in the top right region of the field of view.

Figure 5(a,b) show the cLEDscope image of a binary grid pattern and the node positions for each color. It is apparent that, for the same grid pattern, the detected node positions are different for the red and blue LED illuminations, indicating that our cLEDscope exhibited color-dependent image distortion. The DPC background caused by this image distortion could superimpose over the small DPC signal from the specimen, dramatically degrading the DPC image contrast.

As suggested by Zhang^26^, we acquired ten images as rotating the grid pattern. The node positions in each image were then detected and used to estimate the intrinsic and extrinsic parameters, along with the distortion coefficients. These parameters were used for image distortion correction (see Methods and Materials). Shown in Fig. 5(c,d) are the images of the grid pattern and the detected node positions after applying the distortion correction. It can be seen that the node positions in each color are well co-located. The mean distances between ideal (or distortion-free) and measured nodes, in pixels, were improved from 7.96 to 0.62 in red and 7.27 to 1.06 in blue colors.

**Figure 6:**
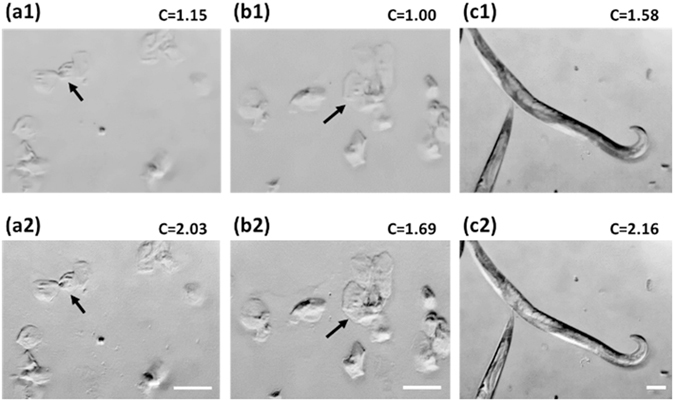
Comparison of distortion-corrected and -uncorrected DPC images of (**a,b**) human epithelial cheek cells and (**c**) *C. elegans*. (**a1–c1**) and (**a2–c2**) are the DPC images without and with distortion correction, respectively. The scale bar denotes 100 μm.

The improved DPC image contrast via the correction technique is demonstrated in Fig. 6. A monolayer of human epithelial cheek cells and *Caenorhabditis elegans (C. elegans)* were imaged using our mobile cLEDscope. Figure 6 shows a comparison of the DPC images acquired without and with the distortion correction. It can be seen that application of the distortion correction scheme improves the DPC image contrast dramatically. In some regions in the distortion-uncorrected DPC image, the spatially varying DPC background, which is due to the distortion, overwhelms the small DPC information of the specimen (see Fig. 6, arrows). In contrast, the small DPC information is clearly visible in the distortion-corrected image. We evaluated the DPC image contrast *C* using the expression $C=({I}_{\max }-{I}_{\min })/{I}_{mean}$ in grayscale (0−255), where ${I}_{\max }$, ${I}_{\min }$, and ${I}_{mean}$ represent the maximum, minimum, and average DPC values over the field of view, respectively. The *C* values for the distortion-uncorrected and -corrected DPC images were measured to be 1.00−1.58 and 1.69−2.16, respectively, indicating contrast improvement by a factor of 1.68−1.77 by use of the distortion-correction algorithm.

### cLEDscope imaging of biological specimens

**Figure 7:**
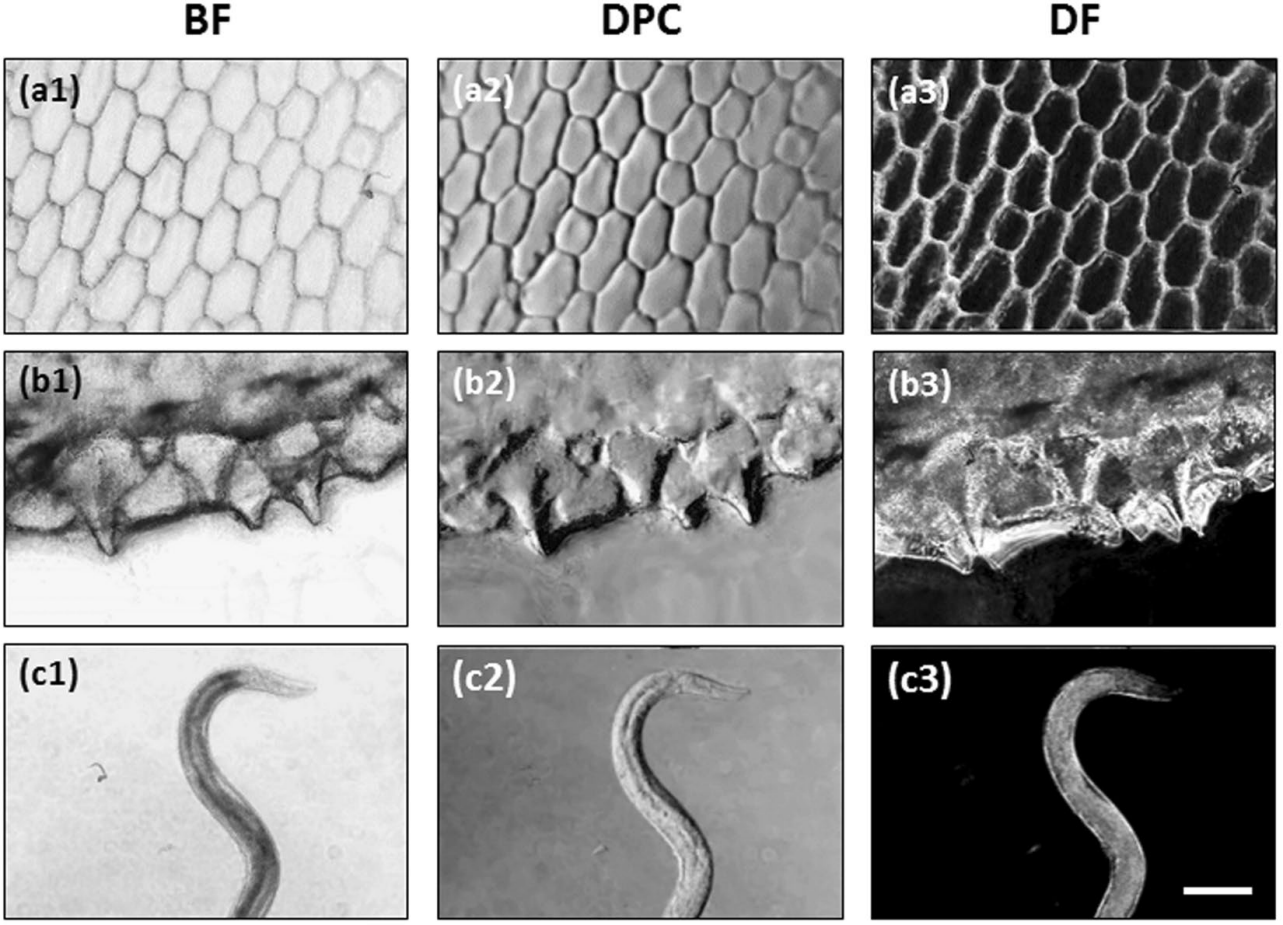
Representative multi-contrast images acquired by mobile cLEDscope. (**a–c**) Images of onion cells, rockfish scales, and *C. elegans*, respectively. The scale bar denotes 200 μm.

We then performed static and dynamic cLEDscope imaging of various biological specimens (Fig. 7). Thin slices of onion cells, the scales of a rockfish, and *C. elegans* were placed on microscope glass slides, which were then loaded in the cLEDscope module for imaging. Figure 7(a) shows BF, DPC, and DF images of the onion cells acquired in a single shot using the mobile cLEDscope. The distinctive shapes of the onion cells are clearly visualized. The three images represent the structural information of the onion cells in different contrasts; however, it is apparent that structural details are most pronounced in the DPC image. The DF image, on the other hand, yields clearer delineation of the cell boundaries. The same observations can also be made for the fish-scale images (Fig. 7(b)). Figure 7(c) shows snapshot images obtained via time-lapse cLEDscope imaging of *C. elegans*. To obtain these images, *C. elegans* immersed in a colon bacterium batch were placed into an imaging chamber (631021, Grace bio-labs, Bend, Oregon, USA) on a microscope slide, and loaded into the cLEDscope module. The internal structures of the *C. elegans* can be observed clearly in the resultant images (Fig. 7(c)). Supplementary video 2 shows dynamic movement of the *C. elegans* recorded by the mobile cLEDscope.

## Methods and Materials

### cLEDscope module fabrication

For mobile cLEDscope, the patterned color LED array was constructed on a custom-designed printed circuit board (PCB). The circular pattern was divided in half and allocated to the red and blue LEDs, whereas the green LEDs were mounted along the perimeter of the circular pattern. Micro-LEDs were employed for each colored illumination for small form factor, and mounted onto the planar PCB board (Blue: SP-190DBN; Green: SP-190DSG; Red: SP-190UHR; HSUKWANG, China). Note that the domed LED arrangement, as demonstrated by Phillips *et al*.^14^, may also be implemented, which would provide higher light efficiency than the planar LED arrays. The circular LED panel was 80 mm in diameter, which matched to the detection NA in our system. The LED array was positioned at 78 mm away from the specimen stage, and was powered by a 3.7-V Li-ion battery.

The specimen stage was designed to load a 1“x3” microscope glass slide. The stage was connected with four steel rods (50 mm in length, 4 mm in diameter), which can slide through the four custom-built slide bushes for lateral positioning of the stage (Supplementary Fig. S1). The assembly was mounted onto a miniature dovetail stage with a focusing knob (DT12, Thorlabs, USA) for axial displacement of the stage. (Fig. S1) Right behind the specimen stage, the achromatic doublet was used as the objective. The pair of the doublet and smartphone built- in lens formed a 3-*f* imaging system.

The module was designed using SolidWorks (Solidworks 2015, Massachusetts, USA) and fabricated with polylactic acid (PLA) using a 3D printer (Wiiboox ONE, JOC, Nanjing, China). The cLEDscope module had dimensions of 84 × 110 × 175 mm^3^.

### Image distortion correction

Camera calibration and image distortion correction in the mobile cLEDscope were performed based on the procedures as described in ref. 26. Consider an imaging configuration, in which a feature point in an object, represented by the coordinate ${\bf{P}}={[x,y]}^{T}$, is imaged via the cLEDscope onto an image feature point, ${\bf{p}}={[u,v]}^{T}$. Defining the corresponding augmented vectors $\mathop{{\bf{P}}}\limits^{{\boldsymbol{\breve{}}}}$ and $\mathop{{\bf{P}}}\limits^{{\boldsymbol{\breve{}}}}$ by adding 1 as the last column: $\mathop{{\bf{P}}}\limits^{{\boldsymbol{\breve{}}}}={[u,v,1]}^{T}{\rm{and}}\mathop{{\bf{P}}}\limits^{{\boldsymbol{\breve{}}}}={[x,y,1]}^{T}$, the relationship between object and image feature points can be expressed as^26^:

4 $$\mathop{{\bf{P}}}\limits^{{\boldsymbol{\breve{}}}}=s{\bf{M}}[{\bf{R}},{\bf{T}}]\mathop{{\bf{P}}}\limits^{{\boldsymbol{\breve{}}}}$$

with

$${\bf{M}}=[\begin{array}{ccc}\alpha  & \gamma  & {u}_{0}\\ 0 & \beta  & {v}_{0}\\ 0 & 0 & 1\end{array}]$$

Here, $s$ denotes a scale factor, and $[{\bf{R}},{\bf{T}}]$, referred to as the extrinsic parameter, represents the rotation and translation relating object and camera pixel coordinate systems. The matrix ${\bf{M}}$ is termed as the camera intrinsic matrix, and is expressed with $({u}_{0},{v}_{0})$ principal point coordinates, $(\alpha ,\beta )$ the scale factors along the $u$ and $v$ axes, and $\gamma$ the skewness of two axes. Note that the same notations in ref. 26 are used.

In order to estimate the intrinsic and extrinsic camera parameters, multiple feature points in the multiple images are recorded, and estimation of the parameters are performed by minimizing an algebraic distance between ideal and recorded pixel coordinates of the feature points. To be specific, for $m$ feature points in $n$ images, the camera parameters are obtained by minimizing the functional:

5 $$\sum _{i=1}^{n}\sum _{j=1}^{m}{\Vert {{\bf{p}}}_{ij}-\bar{{\bf{p}}}({\bf{M}},{{\bf{R}}}_{i},{{\bf{T}}}_{i},{{\bf{P}}}_{j})\Vert }^{2}$$

where $\bar{{\bf{p}}}({\bf{M}},{{\bf{R}}}_{i},{{\bf{T}}}_{i},{{\bf{P}}}_{j})$ is the image of point ${{\bf{P}}}_{j}$ in image $i$. Eq. (5) is a nonlinear minimization problem, which can be readily solved with the Levenberg-Marquardt algorithm.

Once the camera parameters are obtained, the estimation of radial distortion is performed^27^. Assuming $(u,v)$ and $(\bar{u},\bar{v})$ are the ideal (or distortion-free) and distorted pixel image coordinates, the radial distortion can be described as:

6 $$\begin{array}{c}\bar{u}=u+(u-{u}_{0})[{K}_{1}{r}^{2}+{K}_{2}{r}^{4}]\\ \bar{v}=v+(v-{v}_{0})[{K}_{1}{r}^{2}+{K}_{2}{r}^{4}]\end{array}$$

where $r$ denotes the radial distance relative to the principal points, and ${K}_{1}$ and ${K}_{2}$ are the coefficients of the radial distortion, respectively. One can see that, with multiple recorded feature points and camera parameters obtained in the calibration procedure (Eq. 5), ${K}_{1}$ and ${K}_{2}$ can be obtained through linear least-square method. After ${K}_{1}$ and ${K}_{2}$ are obtained, one can further refine the estimates of the parameters by solving Eq. (5) using the results from Eq. (6). Estimation of the camera parameters and distortion coefficients are performed by solving Eq. 5 and Eq. 6 iteratively until convergence.

We performed the aforementioned operations with the functions in Computer Vision System Toolbox in MATLAB. To obtain the feature points in multiple images, we prepared a two-dimensional planar binary grid pattern (or checkerboard pattern) and acquired images with red or blue LEDs only. Ten images were acquired for each color, as rotating the grid pattern. We used ‘detectCheckerboardPoints’ function to detect node positions in the images. The obtained node positions were re-examined and corrected manually, if necessary. The camera parameters were then extracted through the minimization of the above functional, and used to correct for the image distortion.

### cLEDscope Android application

We developed cLEDscope application software (App) that operates on the Android platform. Android provides several camera access and processing functions in Java, the primary programming language for Android applications. However, these functions mainly utilize central processing units (CPUs), and it is difficult to achieve robust image processing in real time using these functions only, because of the limited resources of mobile devices. To resolve this issue, we developed OpenCL-based functions implemented in C++ that run on graphics processing units (GPUs). These functions were used in App via the Java Native Interface (JNI), which allows functions written in languages other than Java to be called.

App primarily provides two camera access modes: image and video. In the image mode, it is possible to capture an image with the maximum resolution supported by the camera. However, a long period of time is required for each image acquisition; therefore, this mode is unsuitable for real-time image processing. In the video mode, each image can be obtained sufficiently rapidly to enable real-time processing; however, the image resolution is smaller than that in the image mode. In our case, the image and video modes were used for the image capturing function and for the display and video recording functions, respectively.

The overall process can be summarized as follows:

1. An image is obtained by the camera;
2. The image is copied to the GPU-accessible graphics memory for computation;
3. As the image obtained in the video mode is in the YUV color space (specifically, NV21), it is converted to the RGB color space;
4. BF, DPC, and DF images are computed via Eqs (1–3), respectively;
5. The data for each image are transferred to the CPU-based memory.

Our experiment was performed on a Samsung Galaxy S5 with the Android 6.0.1 operating system (OS). The camera is equipped with high-performance GPU units and a 16-megapixel rear camera that supports acquisition of images with resolutions of up to 5312 × 2988 and 1920 × 1080 pixels in the image and video mode, respectively. In our case, we used a resolution of 1280 × 720 pixels with a digital zoom of 4x in the video mode to achieve higher throughput. Using the aforementioned optimized procedure, we could achieve processing speeds of up to 14 images per second in the multi-contrast mode (i.e., displaying all BF, DPC, and DF images) and up to 18 images per second in the single-image modes (i.e., a BF, DPC, or DF image).

## Conclusions

We have presented a portable multi-contrast microscope operating on a smartphone platform. Based on color-multiplexing of illumination angles, the microscope enables acquisition of BF, DF, and DPC images in a single shot. Note that implementation of cLEDscope in a smartphone necessitates equal acceptance of red and blue LED light over the entire field-of-view in the specimen plane. For a given built-in lens in the image sensor, we evaluated several inexpensive miniature objectives that provided minimized aberrations for our microscopic imaging. We also devised a field correction algorithm to resolve the chromatic image field distortion. Using the custom-built smartphone application “App,” we demonstrated the static and dynamic multi-contrast imaging capability of our method for various material and biological specimens.

One of the prominent features of our method is its simplicity and robustness. The demonstrations conducted herein employed an inexpensive LED array as a light source, which does not require synchronization with the image acquisition process. This feature is distinct from the multi-contrast microscope described by Phillips *et al*.^14^ and facilitates simple and robust operation. We also utilized a miniaturized objective lens that is inexpensive and commercially available. Note that the imaging performance can be improved through use of a custom-designed lens that further corrects the chromatic and spherical aberrations. This approach is attractive, as, once an appropriate design is obtained, the lenses can be manufactured at high volume via low-cost manufacturing techniques such as imprinting technology. Further, the real-time visualization of multi-contrast images achieved using the proposed method suggests that the computational resources of state-of-the-art smartphones are sufficient for the level of computation required for cLEDscope imaging.

Our prototype operates on a smartphone, utilizing the smartphone internal optics and image sensors. In order to facilitate its adoption for various smartphones and commercialization, a stand-alone cLEDscope module that can operate with diverse mobile devices through the standardized ports (e.g., the USB port) and functions can be developed. This stand-alone module may comprise an LED array, miniature objective and tube lenses, and a color image sensor with wired or wireless connectivity. This stand-alone device is expected to have many applications as an educational and portable diagnostic device.

Further, we envision the realization of fluorescence imaging capability through the addition of appropriate filters. DF illumination could be utilized for excitation and filters could be placed in front of the image sensor. The ability to measure fluorescent molecules would enhance the functionality and applicability of cLEDscope, allowing this method to provide additional biochemically specific information on specimens. These features would allow various cell-based assays to be conducted on a smartphone, providing a new route for the development of micro total-assay systems.

## Electronic supplementary material

Supplementary Video 1

Supplementary Video 2

Supplementary information
