# Supplementary material for: Smartphone-based multi-contrast microscope using color-multiplexed illumination
Source: Sci Rep. 2017 Aug 8;7:7564. doi: 10.1038/s41598-017-07703-w (PMC5548908; doi:10.1038/s41598-017-07703-w)
Supplement: Supplementary file 3 — Supplementary information [file 41598_2017_7703_MOESM3_ESM.doc]

**Supplementary Information**

**Smartphone-based multi-contrast microscope
using color-multiplexed illumination**

Author: Daeseong Jung^1^, Jun-Ho Choi^2^, Soocheol Kim^1^, Suho Ryu^1^, Wonchan Lee^1^, Jong-Seok Lee^2^ and Chulmin Joo^1*^

^1^ Yonsei University, School of Mechanical Engineering, Seoul, 03722, Republic of Korea

^2^ Yonsei University, School of Integrated Technology & Yonsei Institute of Convergence Technology, Incheon, 21983, Republic of Korea

* [cjoo@yonsei.ac.kr](mailto:cjoo@yonsei.ac.kr)


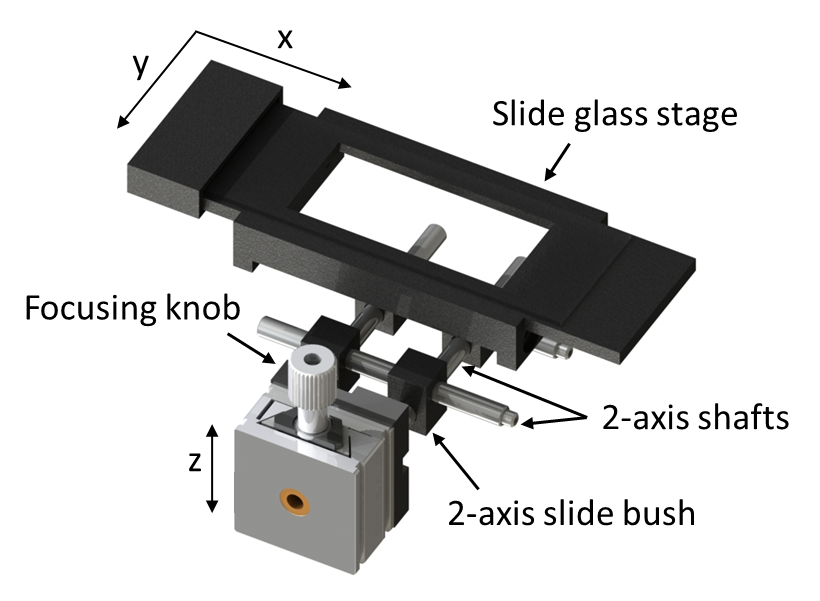


Figure S1. Specimen stage configuration. The stage is designed to load a conventional 1’’x3’’ microscope slide. It is connected with four shafts, which slide through slide bushes for two-dimensional displacement of the stage. The assembly was mounted onto a one-dimensional translation stage with a focusing knob for axial scanning.

**Table S1**. Specification of the evaluated miniature objective lenses

|  | Singlet | Triplet | Doublet |
| --- | --- | --- | --- |
| **Type** | Aspheric | Achromatic | Achromatic |
| **Effective focal length (mm)** | 8 | 10 | 7.5 |
| **Outer diameter (mm)** | 10 | 6.25 | 5 |
| **Vendor** | Thorlabs | Edmund optics | Thorlabs |
| **Model #** | AL108 | #67-417 | AC050-008 |

**Supplementary Video 1.** Operation of smartphone-based mobile cLEDscope. Multi-contrast imaging of rockfish scales was performed with the mobile cLEDscope.

**Supplementary Video 2.** Dynamic multi-contrast imaging of *C. elegans*
